# Comparative genomic analysis of plasmids encoding metallo-β-lactamase NDM-5 in Enterobacterales Korean isolates from companion dogs

**DOI:** 10.1038/s41598-022-05585-1

**Published:** 2022-01-28

**Authors:** Su Min Kyung, Sung-Woon Choi, Jaewon Lim, Soojin Shim, Suji Kim, Young Bin Im, Na-Eun Lee, Cheol-Yong Hwang, Donghyuk Kim, Han Sang Yoo

**Affiliations:** 1 Department of Infectious Disease, College of Veterinary Medicine, Seoul National University, Seoul, Republic of Korea; 2 School of Life Sciences, Ulsan National Institute of Science and Technology (UNIST), Ulsan, 44919 Republic of Korea; 3 Department of Veterinary Dermatology, College of Veterinary Medicine, Seoul National University, Seoul, Republic of Korea; 4 Present Address: Department of Mechanical and Biofunctional Systems, Institute of Industrial Science, University of Tokyo, Tokyo, 153-8505 Japan

**Keywords:** Microbiology, Health care, Medical research

## Abstract

Carbapenems are broad-spectrum antibiotics widely used for the treatment of human infections caused by multidrug-resistant (MDR) Gram-negative bacteria. However, emerging carbapenemase-producing Enterobacterales (CPE) are rising as a public threat to human and animal health. We screened clinical bacterial isolates from 241 dogs and 18 cats hospitalized at Veterinary Medical Teaching Hospital, Seoul National University, from 2018 to 2020 for carbapenemase production. In our study, 5 strains of metallo-β-lactamase NDM-5-producing *Escherichia coli* and *Klebsiella pneumoniae* were isolated from 4 different dogs. Multilocus sequence typing (MLST) results showed that all *E. coli* strains were ST410 and all *K. pneumoniae* strains were ST378. Whole genome analysis of the plasmid showed that *bla*_NDM-5_ is carried on a IncX3 plasmid, showing a high concordance rate with plasmids detected worldwide in human and animal isolates. The *bla*_NDM_ gene was associated with the *ble*_MBL_ gene and the ISA*ba125* element, truncated with the IS5 element. The results of this study show that CPE has already become as a threat to both animals and humans in our society, posing the necessity to solve it in terms of "One Health". Therefore, preventive strategies should be developed to prevent the spread of CPE in animal and human societies.

## Introduction

Carbapenem is considered the last resort antibiotic for multidrug-resistant (MDR) Gram-negative bacteria. Carbapenemases produced by bacteria can hydrolase antibiotics containing β-lactam rings, including carbapenems, with even higher potential than extended-spectrum β-lactamases (ESBLs)^1^. Among the β-lactamases categorized into four Ambler classes of A-D, there are three classes to which carbapenemases belong, namely, class A, class B and class D^2^. Among class B carbapenemases, New Delhi metallo-β-lactamase (NDM) is known to be more effective than other groups and can be inhibited by metal chelators such as EDTA and mercaptopropionic acid^3^.

The increasing global spread of CPE, including *Klebsiella pneumoniae* and *Escherichia coli,* is considered a public threat to human and animal society, and these bacteria are listed by the World Health Organization (WHO) as priority 1 critical pathogens^4^. *E. coli* is a commensal bacterium colonizing the mucosal layer of the mammalian colon, including that of human infants, within a few hours after birth that can occasionally cause disease in immunocompromised hosts or in those with breached barriers of the gastrointestinal tract, including patients with peritonitis^5^. *K. pneumoniae* is an opportunistic pathogen causing pneumonia, sepsis, UTIs, bacteremia, meningitis and pyogenic liver abscesses, often in immunosuppressed patients via hospital infection^6^. *K. pneumoniae* resides not only in the environment but also on medical devices such as urinary catheters and endotracheal tubes and is frequently disseminated between health care workers and patients in hospitals^7^. Both *E. coli* and *K. pneumoniae* live alongside humans and animals and are emerging as threats as they gain resistance against antimicrobial agents, including resistance against carbapenems.

Since the first report of the isolation of a *K. pneumoniae* NDM-bearing strain from a patient in Sweden in 2009, NDM has spread worldwide due to its location on mobile genetic elements such as plasmids, transposons and integrons^8^. Conjugative plasmids such as Incompatibility group (Inc) F, A/C, L/M, N and X are associated with dissemination of *bla*_NDM_ via horizontal gene transfer (HGT)^2^. In 2011, NDM-5 was first detected in *E. coli* from the United Kingdom with mutations of two amino acid positions of 88 (Val → Leu) and 154 (Met → Leu) from NDM-1 that resulted in increased action against carbapenems^9^. NDM-5 is disseminated worldwide and has been detected in various countries including Australia^10^, Denmark^11^, Italy^12–14^, Switzerland^15^, the Netherlands^16^, China^17^, India^18^, South Korea^19^, and the USA^20^.

Although prescription of carbapenem in animal medicine is prohibited in any part of the world by the World Organisation for Animal Health (OIE), the *bla*_NDM-5_ gene has been detected worldwide not only in companion animals, including dogs and cats^15, 21^, but also in domestic animals^17, 22, 23^. In South Korea, CPE were first reported among *E. coli* of companion animals in 2018 and reported as New Delhi metallo-β-lactamase-5 (NDM-5)^21^. Regarding the increasing threat of CPE, continuous surveillance and genetic characterization of CPE isolates have been required to develop the control measures against their spread in human and animal society.

Based on the current situation of CPE, Korean isolates of CPE from companion animals were characterized phenotypically and genotypically. Also, the genetic characteristics of the isolates were revealed by comparison with those from other countries.

## Results

### Profiles and antimicrobial susceptibility of the isolates

**Figure 1:**
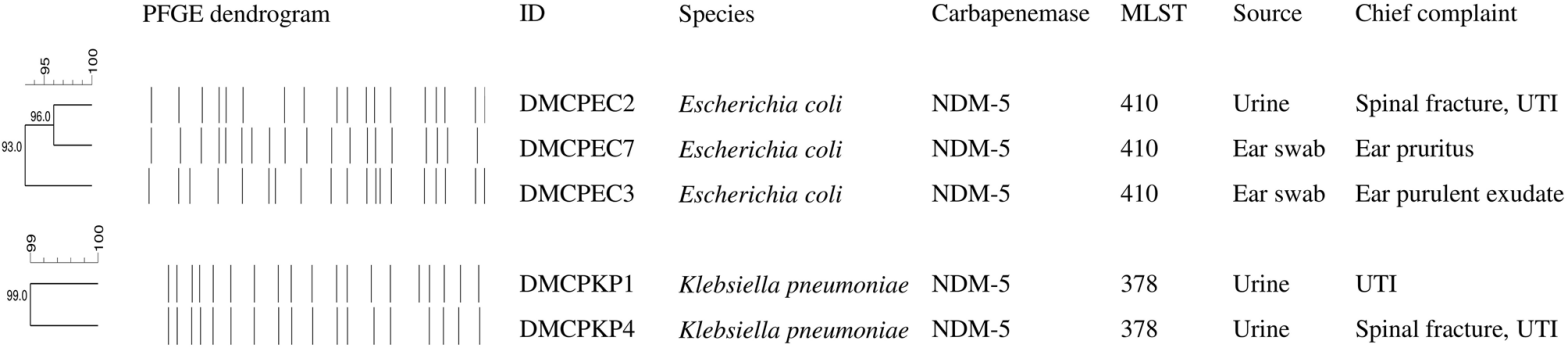
General information of the NDM-5-producing Enterobacterales strains based on patient history, PFGE and MLST data. PFGE patterns were generated and analyzed in Dice similarity coefficient with Unweighted Pair-Group Method with Arithmetic Mean (UPGMA) dendrogram via BioNumerics, version 6.6 (Applied Maths NV, Belgium).

A total of 5 carbapenemase-producing strains were isolated from 520 isolates and identified as 3 *E. coli* strains and 2 K*. pneumoniae* strains. Four strains were isolated in 2019 and one (DMCPEC3) in 2020. Three strains were isolated from urine samples, and the others were isolated from ear swab samples (Fig. 1). According to hospital records, the likelihood of physical contact between patients was low. Notably, one *E. coli* strain (DMCPEC2) and one *K. pneumoniae* strain (DMCPKP4) were isolated from different urine samples from the same dog. Although the dog was suffering from cystitis and receiving antibiotic treatment with enrofloxacin and amoxicillin/clavulanic acid, it did not show any improvement of clinical signs. The other strain of *K. pneumoniae* (DMCPKP1) was isolated from urine specimen of a dog patient with increased urine volume and odor. The patient was treated with amoxicillin/clavulanic acid and enrofloxacin but showed no improvement. The other 2 *E. coli* isolates (DMCPEC3, DMCPEC7) were isolated from ear swab samples of 2 different dogs. Interestingly, the two dogs had previously been treated in the same facility in Seoul, but the timing of each individual's visit was unknown. DMCPEC3 was isolated from a dog with ear pruritus in 2020, but has never been applied with antibiotics in our facility. DMCPEC7 was isolated from ear purulent exudate of other dog patient, which was treated with enrofloxacin.

All 5 isolates showed resistance not only to carbapenems, including imipenem (> 256 µg/ml), meropenem (32–128 µg/ml), and ertapenem (32–64 µg/ml) (Table 1), but also to various other antibiotics. Resistant phenotypes included resistance to cefotaxime, ceftazidime, ceftriaxone, gentamicin, ampicillin, levofloxacin, norfloxacin, ofloxacin, nalidicic acid and tetracycline. However, all isolates were susceptible to polymyxin B and colistin. As for aztreonam and tobramycin, *E. coli* strains appeared susceptible to intermediate, while *K. pneumoniae* strains showed resistance. The resistant phenotypes were determined referring to the CLSI clinical interpretation breakpoints.

**Table 1:** Minimum inhibitory concentration level of 5 carbapenemase-producing strains against various antibiotics (μg/mL).

Antibiotic class	Antimicrobial agents	Minimum inhibitory concentration (μg/mL)				
		*E. coli* (DMCPEC2)	*E. coli* (DMCPEC3)	*E. coli* (DMCPEC7)	*K. pneumonia* (DMCPKP1)	*K. pneumonia* (DMCPKP4)
Carbapenems	Ertapenem	64	32	64	64	64
	Imipenem	> 256	> 256	> 256	> 256	> 256
	Meropenem	64	32	32	128	128
1st Cephalosporins	Cephradine	> 256	> 256	> 256	> 256	> 256
3rd Cephalosporins	Cefotaxime	> 256	> 256	> 256	> 256	> 256
	Ceftazidime	> 256	> 256	> 256	> 256	> 256
	Ceftriaxone	> 256	> 256	> 256	> 256	> 256
4th Cephalosporins	Cefepime	> 256	> 256	> 256	32	24
Aminoglycosides	Amikacin	8	8	4	> 256	> 256
	Gentamicin	96	96	48	> 256	> 256
	Kanamycin	32	64	32	> 256	> 256
	Kanamycin B	8	8	4	> 256	> 256
	Neomycin	4	2	2	1	1
	Streptomycin	> 256	> 256	> 256	8	4
	Tobramycin	6	4	4	> 256	> 256
Aminopenicillins	Ampicillin	> 256	> 256	> 256	> 256	> 256
	Amoxicillin	> 256	> 256	> 256	> 256	> 256
Phenicols	Chloramphenicol	16	64	16	> 256	> 256
	Florfenicol	16	4	16	> 256	> 256
Folate pathway inhibitors	Trimethoprim	0.5	> 256	0.5	> 256	> 256
Fluoroquinolones	Enrofloxacin	256	> 256	> 256	> 256	> 256
	Levofloxacin	64	128	64	64	64
	Norfloxacin	> 256	> 256	> 256	> 256	> 256
	Ofloxacin	128	256	128	128	128
Glycopeptides	Polymyxin B	≤ 2	≤ 2	≤ 2	≤ 2	≤ 2
	Colistin	0.19	0.5	0.19	0.19	0.5
Macrolides	Erythromycin	> 256	> 256	> 256	> 256	> 256
Monobactams	Aztreonam	8	3	3	24	24
Natural Penicillins	Penicillin G	> 256	> 256	> 256	> 256	> 256
Oebucukkubase	Oxacillin	> 256	> 256	> 256	> 256	> 256
Quinolones	Nalidixic acid	> 256	> 256	> 256	> 256	> 256
Sulfonamides	Sulfamethoxazole	> 256	> 256	> 256	> 256	> 256
Tetracyclines	Tetracycline	> 256	256	> 256	> 256	> 256
	Tigecycline	0.125	0.25	0.064	0.38	0.5
Ureidopenicillins	Azlocillin	> 256	> 256	> 256	> 256	> 256

Either broth or E-test methods were performed following recommendation of Clinical and Laboratory Standards Institute (CLSI) interpretation criteria^48^. E-test methods were used for 8 antimicrobial agents: ceftazidime, ceftriaxone, cefepime, gentamicin, tobramycin, colistin, aztreonam, tigecycline. The others were tested via broth microdilution method. *E. coli* strain ATCC 25922 was used as a quality control strain. CPE isolates showed resistance imipenem (> 256 μg/ml), meropenem (32 ~ 128 μg/ml), and ertapenem (32 ~ 64 μg/ml) while seeming to be susceptible against polymyxin B, colistin, and tigecycline.

PCR amplification followed by sequencing revealed that all 5 strains of carbapenemase-harboring strains produced NDM-5, with two amino acid mutations at positions 88 (Val to Leu) and 154 (Met to Leu), regardless of bacterial species or source of isolation. The other four major carbapenemase (*bla*_KPC_, *bla*_VIM_, *bla*_IMP_, and *bla*_OXA-48_) markers were not detected in all isolates.

### Genotypic relatedness according to MLST and PFGE

The genotypic relationships between isolated strains determined with MLST revealed that all *E. coli* strains belonged to ST410 and all *K. pneumoniae* strains belonged to ST378. The epidemiological similarity results between PFGE indicated high relatedness of the *E. coli* strains (> 93%) and the *K. pneumoniae* strains (99%).

### Conjugation transferability and plasmid analysis

Conjugation assays confirmed the transferability of the *bla*_NDM-5_ gene in broth mating at frequencies of 1.0 × 10^−4^ to 1.0 × 10^−5^ (Table 2). All transconjugants were confirmed by PCR identification. In conjugation assay, transferability was proven not only in *E. coli* but also in the case where *K. pneumoniae* strains contributed as donors.

**Table 2:** Genomic information of plasmids harboring *bla*_NDM-5_ gene in Carbapenemase-producing Enterobacterales isolates in this study.

ID	Species	Plasmid size (bp)	Accession numbers	Conjugation frequency (T/D)	CDS number	GC contents (%)
DMCPEC2	*Escherichia coli*	46,288	MW415440	$1.06\times {10}^{5}$	60	46.7
DMCPEC3	*Escherichia coli*	45,805	MW415441	$2.50\times {10}^{4}$	60	46.6
DMCPEC7	*Escherichia coli*	45,594	MW415442	$1.33\times {10}^{5}$	60	46.6
DMCPKP1	*Klebsiella pneumoniae*	45,311	MW415443	$1.61\times {10}^{5}$	61	46.9
DMCPKP4	*Klebsiella pneumoniae*	45,311	MW415444	$5.84\times {10}^{5}$	61	46.9

Plasmid DNAs were isolated with the QIAGEN Plasmid Mini Kit (QIAGEN, Germany) and sequenced with the Illumina MiSeq sequencing platform.

Complete sequences of all five NDM-5 harboring plasmids were obtained using the Illumina MiSeq Sequencing System. Plasmid sequencing and PCR replicon typing results identified all isolated plasmids as IncX3, with lengths varying from 45–46 kb, GC contents varying from 46.5 to 46.91% and 60–61 CDSs (Table 2). Downstream of *bla*_NDM-5_, the *ble*_MBL_ gene encoding the bleomycin-resistant protein, the *trpF* gene encoding phosphoribosylanthranilate isomerase, the *dsbC* gene for oxidoreductase, the cutA gene fragment encoding the periplasmic divalent cation tolerance protein inserted by the *IS26* element and the truncated *umuD* gene encoding a mutagenesis protein were identified.

### Comparative plasmid genome analysis

**Figure 2:**
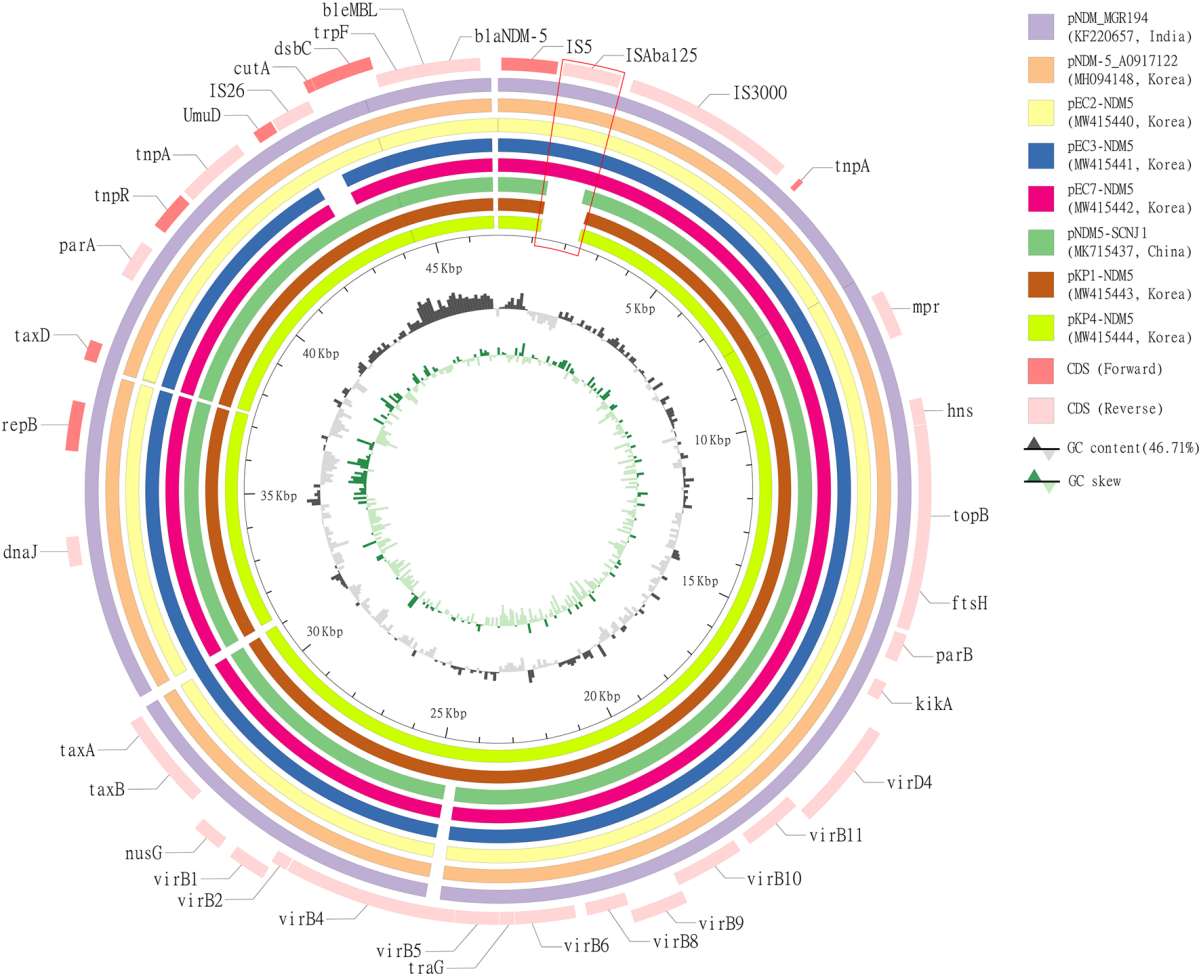
Schematic map of comparative circular genome structure analysis of 8 IncX3 plasmids. Circular maps were used to illustrate and compare the backbone and the location of the genetic load region of plasmids. GC skew was featured based on data of pKP4-NDM5. Genome alignments were performed by Mauve^24^, and the circular map was generated with CIRCOS (http://circos.ca/).

**Figure 3:**
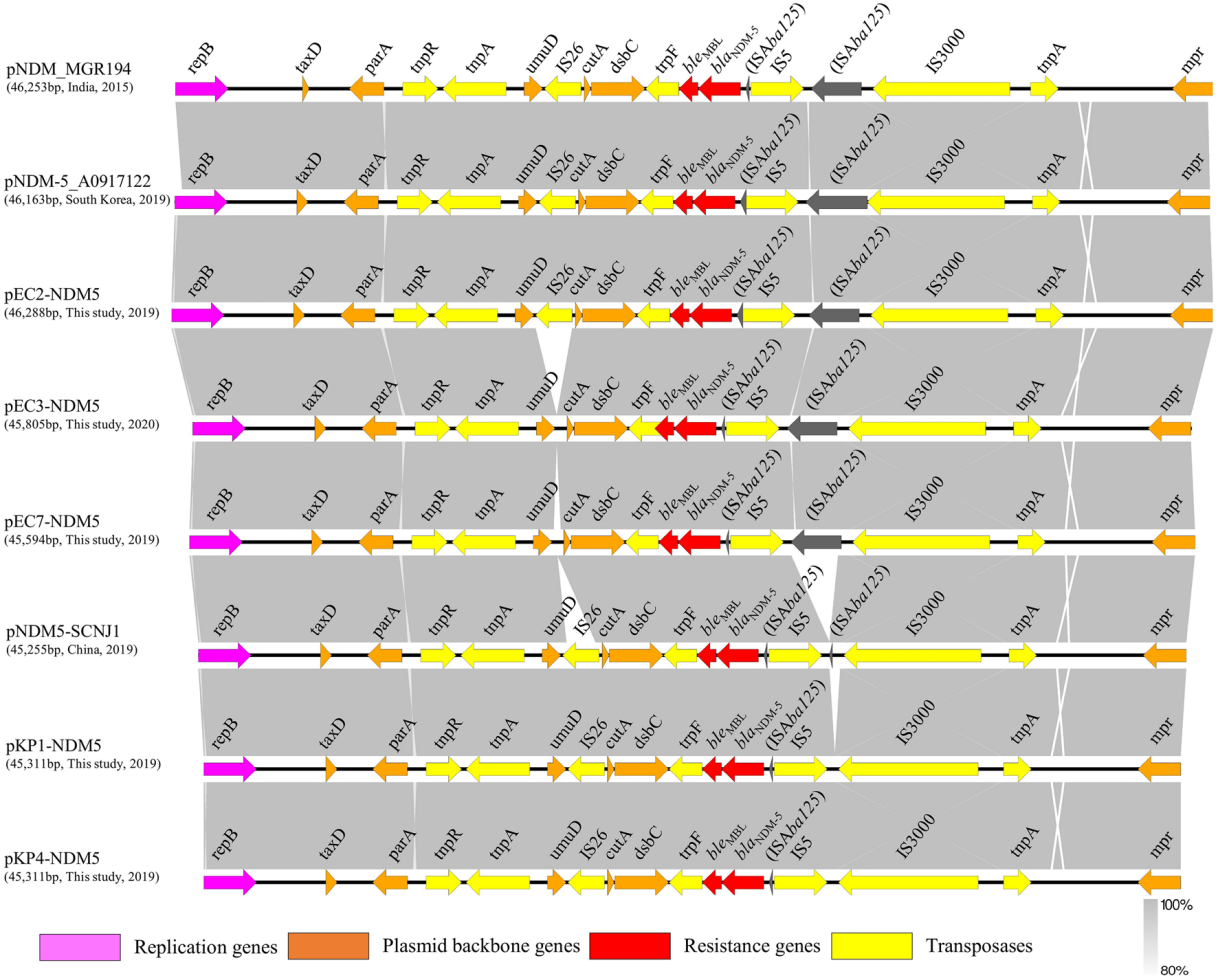
Genetic load sequence context of IncX3 *bla*_NDM–5_ plasmids. Genes are denoted by arrowheads and colored based on class of gene function, sorting by replication, transposon, antimicrobial resistance genes and plasmid backbone elements. Gray shades denote shared regions with a high degree of homology. Easyfig 2.2.3 (https://github.com/mjsull/Easyfig/wiki) was used for this pairwise BLASTn alignment comparing analysis^25^. The accession numbers were: pNDM_MGR194 (KF220657.1); pNDM-5_A0917122 (MH094148); pEC2-NDM5 (MW415440); pEC3-NDM5 (MW415441); pEC7-NDM5 (MW415442); pNDM5-SCNJ1 (MK715437.1); pKP1-NDM5 (MW415443); pKP4-NDM5 (MW415444).

The genetic load regions and the whole circular sequences of the five plasmids were compared with three reference IncX3 plasmids from three different countries (pNDM-5_A0917122 from South Korea, pNDM_MGR194 from India and pNDM5-SCNJ1 from China) (Figs. 2, 3). pEC2-NDM5 showed high identity and similar gene features with two reference plasmids, pNDM-5_A0917122 and pNDM_MGR194, isolated from our country and India, respectively. pNDM-5_A0917122 was the plasmid isolated from companion animal of South Korea, while pNDM_MGR194 was isolated from human patient of India. On the other hand, the middle parts of transposon IS26 in pEC3-NDM5 and pEC7-NDM5 were lost over a length of 567 base pairs. Plasmids from *K. pneumoniae* showed higher identity with pNDM5-SCNJ1 from human sputum of China, which was also detected from isolates of *K. pneumoniae*. The ISA*ba125* region was previously found to be truncated (166 bp upstream from the *bla*_NDM-5_ start codon) by the IS5 element, with 67 base pairs of remnants between the *bla*_NDM-5_ and IS5 elements^26^. While the ISA*ba125* region between the IS5 element and IS3000 element was found to be shortened by 112 base pairs in pNDM5-SCNJ1 compared with other IncX3 plasmids, the same gene area of *K. pneumoniae* in our study was completely lost. Two plasmids from *K. pneumoniae* strains, pKP1-NDM5 and pKP4-NDM5, showed 100% identity to each other.

## Discussion

This investigation identified Enterobacterales bearing NDM-5-producing IncX3 from companion animals. This was the first report in our country of *K. pneumoniae* as a carbapenemase producer from companion animals, whereas the presence of carbapenemase-producing *E. coli* was reported previously in our country^21^. Of note, two different isolates, one *E. coli* (DMCPEC2) and one *K. pneumoniae* (DMCPKP4) came from different urine samples collected from a single dog at different times. Their identical carbapenemase and plasmid features (99.85% identity) likely indicate the possibility of bacterial interspecies horizontal transfer of genetic elements inside the host, or on medical devices such as urinary catheter. Sporadic dissemination among companion animals or in animal hospitals is suspected, regarding the identical carbapenemase gene profiles, the identical genetic environment structures, the same MLST types and PFGE patterns showing the same pulsotype (> 93% along *E. coli* strains and > 99% along *K. pneumoniae* strains), and the genetic environment structure (ISA*ba125*-*bla*_NDM-5_-*ble*_MBL_-trpF-dsbC-cutA-umuD).

Plasmids can transport multiple antibiotic resistance genes (ARGs) through conjugation between heterogeneous species as well as the identical species, which makes plasmids crucial for bacterial colonization and virulence potential^27^. Incompatibility group IncX3 plays a major role in dissemination of antibiotic resistance genes and is known to harbor *bla*_NDM-1_ and *bla*_NDM-5_ rather than other *bla*_NDM_ variants^2^. IncX3 plasmids carrying various carbapenemase genes seem to be disseminated worldwide, mainly in China^28^. A recent investigation performed in China revealed that the IncX3 plasmid harboring *bla*_NDM-5_ is disseminated among Enterobacterales in both humans and animals^22, 29^. In animals, IncX3 plasmids carrying *bla*_NDM_ have been detected in *E. coli* from swine and chickens in various regions in China^17, 30^. The identification of IncX3 from China and South Korea is not a surprise, considering the global dissemination of these plasmids. Among carbapenemase-harboring plasmids, the IncX3 plasmid is also dominant in South Korea, showing dissemination within diverse bacterial species, followed by IncFII and IncA/C^19^. *bla*_NDM_ has been suggested to be disseminated from *Acinetobacter baumannii* to Enterobacterales via the mobile gene element IS*Aba125*^31^. All five isolates in our study support this idea, with association of transposon elements of the upstream IS*Aba125* element. As described and predicted previously, the IS*Aba125* element was lost in *K. pneumoniae* strains examined in our study via horizontal transfer or host response mechanisms^29^.

*E. coli* ST410 is known for interspecies transmission along people, environment, wildlife and companion animals^32, 33^. Additional isolation of *bla*_NDM-5_ from *E. coli* ST410 in our country is posing threat of human infection from companion animal or vice versa. A recent study from Denmark suggested *E. coli* ST410 as a new high-risk clone, causing recurrent infections such as bloodstream infections and carrying carbapenemases such as NDM-5 or OXA-181^34^. *E. coli* ST410 is reported as NDM-5 producer in various countries worldwide, including China^35^, the United Kingdom^36^ and South Korea^21^. In South Korea, ST410 is the 3^rd^ most dominant NDM-producing clinical *E. coli* strain, which includes reports from companion animals^19, 21, 37^. Therefore, detailed investigation to discover the role and dissemination of *E. coli* ST410 in our country is necessary. To prevent public outbreak, infection control across people, environment, wildlife and companion animal based on One Health Approach is needed.

*K. pneumoniae* ST378 has never been reported as CPE, even though NDM-5-producing *K. pneumoniae* has been reported worldwide from various strains^16, 23, 38–42^. While the predominant NDM-producing clinical *K. pneumoniae* strain is ST1061 in South Korea^19^, ST378 was reported as a common sequence type among ESBL- and/or AmpC β-lactamase-producing clinical *K. pneumoniae* isolates in Taiwan^43^. However, information on these clones of ST378 is still scarce, and further monitoring investigations are needed to avoid additional dissemination in our country. Considering that the plasmid of this new strain is similar to the previously described ones, it should be considered as additional evidence of horizontal spread of carbapenemase harboring IncX3 plasmids between Enterobacterales, discovered from companion dog hosts.

While the transmission route of these CPE isolates is still unclear, there are several possible hypotheses. First is circulation inside veterinary teaching hospital environment, considering genetic similarity between CPE strains and their urinary tract infection (possibly urinary catheter infection). Transmission via physical contact of companion animals is also considerable, regarding that MLST types of carbapenemase-harboring *E. coli* isolates discovered in companion animals in our country are identical, as ST410^21^. Also considering that these isolates were from a tertiary referral hospital in South Korea, it could be indicating unauthorized usage of carbapenems in local veterinary hospitals in our country. Interestingly, two of the host dogs had previously visited the same animal hospital, but it was not known whether they had been in contact, and this information alone could not reach a definite conclusion. Lastly, it could be acquired from contaminated feed or drinking water, or from contact with their human owners.

In this study, the CPE strains were isolated from clinical lesions, and each strain caused ear and urinary tract clinical signs in the host dog. Therefore, the possibility of these strains to be infected and become human clinical pathogens should be considered. Also, bacterial screening of human owner is required to figure out the possibility of human-to-animal transmission, or vice versa.

In conclusion, additional emergence of CPE in this study shows the dissemination of carbapenemase in our society, which is already a public concern considering the forbidden usage of carbapenems in animals. Therefore, further investigation is necessary to unveil the role of IncX3 plasmids carrying *bla*_NDM-5_ and the evidence of transmission between human owners and companion animals.

## Methods

### Bacterial strain identification and carbapenemase gene detection

Specimens, urine and swabs of skin and ears, were collected from suspected of being infected companion dogs and cats hospitalized at Veterinary Medical Teaching Hospital, Seoul National University, from 2018 to 2020. The specimens were collected by professional veterinarians in accordance with the Guide for the Care and Use of Laboratory Animals and the Animal Welfare Act. From the collected specimens, a total of 520 clinical isolates from 241 dogs and 18 cats were isolated using sheep blood agar (Hangang, Gyeonggi, Korea) for the diagnostic isolation and stored in Tryptic Soy Broth (Thermo Fisher Scientific Oxoid Ltd., Basingstoke, UK) with 50% glycerol at − 70 °C for further epidemiological studies. The stored clinical specimens were subject to screening for carbapenem resistance on CHROMagar mSuperCARBA agar (CHROMagar). Total DNA of surviving isolates was purified by a Wizard Genomic DNA Purification Kit (Promega, Madison, WI) and amplified using PCR specific primers detecting 5 widespread carbapenemase genes (*bla*_KPC_, *bla*_NDM_, *bla*_VIM_, *bla*_IMP_, and *bla*_OXA-48_) using previously designed primers^44–47^. Sequences were identified by the Sanger sequencing method and compared with GenBank data (www.ncbi.nlm.nih.gov/GenBank) with the Basic Local Alignment Search Tool (BLAST) network service. Microbial species were confirmed with matrix-assisted laser desorption ionization–time of flight-mass spectrometry (MALDI–TOF–MS; Bruker Daltonik GmbH, Bremen, Germany) and further confirmed using 16S rRNA sequencing with the primer pair 27F/1492R.

### In vitro antibiotic susceptibility testing

Minimum inhibitory concentrations (MIC) of the isolates were determined for 35 antimicrobial agents: imipenem, meropenem, ertapenem, cephradine, cefotaxime, ceftazidime, ceftriaxone, cefepime, amikacin, gentamicin, kanamycin, kanamycin B, neomycin, streptomycin, tobramycin, ampicillin, amoxicillin, chloramphenicol, florfenicol, trimethoprim, enrofloxacin, levofloxacin, norfloxacin, ofloxacin, polymyxin B, colistin, erythromycin, aztreonam, penicillin G, oxacillin, nalidixic acid, sulfamethoxazole, tetracycline, tigecycline and azlocillin. Either broth microdilution method or Etest (bioMérieux, Marcy L'Etoile, France) strip method on Mueller–Hinton agar (Difco Laboratories, Detroit, MI) were applied depending on availability of antibiotics. E-test methods were used for 8 antimicrobial agents: ceftazidime, ceftriaxone, cefepime, gentamicin, tobramycin, colistin, aztreonam, tigecycline. The others were tested via broth microdilution method. Both methods were performed and interpreted following the recommendation of the Clinical and Laboratory Standards Institute (CLSI) interpretation criteria^48^. *E. coli* strain ATCC 25922 was used as a quality control strain.

### Pulse-field gel electrophoresis and multilocus sequence typing

The genetic relationships between isolated strains were analyzed via pulse-field gel electrophoresis (PFGE) and multilocus sequence typing (MLST). As recommended in the CDC’s protocol for *E. coli*, agarose plugs containing genomic DNA of the three *E. coli* and two *K. pneumoniae* isolates were digested with *XbaI* restriction enzyme and separated for 18 h at 14 °C using a CHEF-Mapper PFGE system at 6 V/cm (Bio-Rad, Hercules, CA)^49^. A lambda ladder (Bio-Rad) was used as a DNA size marker. PFGE patterns were then analyzed, and a Dice similarity coefficient with the unweighted pair-group method with arithmetic mean (UPGMA) dendrogram was generated using BioNumerics, version 6.6 (Applied Maths NV, Belgium). MLST of *E. coli* was performed using seven housekeeping genes (*adk*, *fum*C, *gyr*B, *icd*, *mdh*, *pur*A, and *rec*A) following a standardized protocol as previously described and assigned using an online database (http://mlst.warwick.ac.uk/mlst/dbs/Ecoli)^50^. MLST of *K. pneumoniae* was also performed using seven housekeeping genes (*rpo*B, *gap*A, *mdh*, *pgi*, *pho*E, *inf*B, and *ton*B), and assignment was based on an online database for *K. pneumoniae* (https://bigsdb.pasteur.fr/klebsiella/klebsiella.html)^51^.

### Conjugation assay

A conjugation assay was performed for all of the NDM-harboring isolates with sodium azide-resistant *E. coli* J53 as the recipient strain in a 1:1 ratio in broth. Each 2 mL culture of donor and recipient cells in the logarithmic phase was resuspended in fresh trypticase soy broth (TSB) and mixed before overnight incubation at 30 °C without agitation. Transconjugants were selected on trypticase soy agar (TSA) plates containing both sodium azide (100 μg/ml; Sigma Chemical Co., St. Louis, Mo.) and meropenem (1 μg/ml: Sigma-Aldrich, St. Louis, MO). Species identifications were confirmed using a MALDI-TOF MS Biotyper and 16S rRNA sequencing. The presence of *bla*_NDM_ genes was confirmed by PCR analysis. The conjugation transfer frequency of carbapenemase-producing genes was expressed as transconjugants per donor cell (T/D) following methods previously described^52^.

### Plasmid sequencing and mapping

Plasmid DNA was isolated using a QIAGEN Plasmid Mini Kit (QIAGEN, Germany) and sequenced with the Illumina MiSeq Sequencing System (Illumina, San Diego, CA, USA), generating 300 bp paired-end reads (1 Gbp per sample). FastQC (v.0.11.5) was used for sequence quality analysis, after which the sequences were filtered and trimmed using the program Trimmomatic (v.0.36). SPAdes (v3.13.0) software (https://github.com/ablab/spades)^53^ was utilized for de novo assembly, and Prokka (v.1.10) was used for annotation. To identify antibiotic resistance genes, annotation of coding sequences (CDSs) was performed with bioinformatic tools including the ARG-ANNOT (Antibiotic Resistance Gene-ANNOTation) database, the NCBI Prokaryotic Genome Annotation Pipeline (www.ncbi.nlm.nih.gov/books/NBK174280), and ResFinder (https://cge.cbs.dtu.dk/services/ResFinder/). For comparison, three other related plasmids, namely, pNDM-5_A0917122 (companion animal isolate from South Korea, accession number MH094148), pNDM_MGR194 (human isolate from India, accession number KF220657.1), and pNDM5-SCNJ1 (human isolate from China, accession number MK715437.1), were aligned and interpreted with the BLAST network service. Multiple plasmid alignments were performed by Mauve^24^, and the circular maps of plasmid were generated using CIRCOS (http://circos.ca). Plasmid mapping for genetic load sequence was performed and visualized using Easyfig version 2.2.3 (https://github.com/mjsull/Easyfig/wiki) software^25^.

Incompatibility typing of the *bla*_NDM-5_ plasmid was additionally confirmed from plasmid sequencing results in silico by PCR-based replicon typing^54, 55^. Plasmid nucleotide sequences have been deposited in GenBank with the following accession nos.: MW415440 (pEC2-NDM5), MW415441 (pEC3-NDM5), MW415442 (pEC7-NDM5), MW415443 (pKP1-NDM5), and MW415444 (pKP4-NDM5).

## Data Availability

Publicly available datasets were analyzed in this study. This data can be found in Table 2 for all accession numbers. All data generated or analyzed during this study have been submitted with this manuscript. All genetic information of the plasmids was deposited in GenBank. Therefore, all data from this study are available publically.
